# Inactivation of the von Hippel-Lindau tumour suppressor gene induces Neuromedin U expression in renal cancer cells

**DOI:** 10.1186/1476-4598-10-89

**Published:** 2011-07-26

**Authors:** Sarah K Harten, Miguel A Esteban, Deepa Shukla, Margaret Ashcroft, Patrick H Maxwell

**Affiliations:** 1 Division of Medicine, University College London, London, UK; 2 South China Institute of Stem Cell Biology and Regenerative Medicine, Guangzhou Institute of Biomedicine and Health, Chinese Academy of Sciences, Guangzhou 510663, China; 3 ACRF Centre for Cancer Epigenetics, Queensland Institute of Medical Research, Herston Road, Brisbane, Australia

## Abstract

**Background:**

209 000 new cases of renal carcinoma are diagnosed each year worldwide and new therapeutic targets are urgently required. The great majority of clear cell renal cancer involves inactivation of *VHL*, which acts as a gatekeeper tumour suppressor gene in renal epithelial cells. However how VHL exerts its tumour suppressor function remains unclear. A gene expression microarray comparing RCC10 renal cancer cells expressing either *VHL *or an empty vector was used to identify novel *VHL *regulated genes.

**Findings:**

NMU (Neuromedin U) is a neuropeptide that has been implicated in energy homeostasis and tumour progression. Here we show for the first time that *VHL *loss-of-function results in dramatic upregulation of NMU expression in renal cancer cells. The effect of *VHL *inactivation was found to be mediated via activation of Hypoxia Inducible Factor (HIF). Exposure of *VHL *expressing RCC cells to either hypoxia or dimethyloxalylglycine resulted in HIF activation and increased NMU expression. Conversely, suppression of HIF in *VHL *defective RCC cells via siRNA of HIF-α subunits or expression of Type 2C mutant *VHL*s reduced NMU expression levels. We also show that renal cancer cells express a functional NMU receptor (NMUR1), and that NMU stimulates migration of renal cancer cells.

**Conclusions:**

These findings suggest that NMU may act in an autocrine fashion, promoting progression of kidney cancer. Hypoxia and HIF expression are frequently observed in many non-renal cancers and are associated with a poor prognosis. Our study raises the possibility that HIF may also drive NMU expression in non-renal tumours.

## Findings

Kidney cancer is responsible for 102 000 deaths per year worldwide and prognosis is generally poor [1]. Clear cell renal cell carcinoma (CCRCC) is the commonest form of kidney cancer and the von Hippel-Lindau (*VHL*) tumour suppressor gene is mutated or inactivated in the vast majority of these tumours [2]. Mutations in *VHL *also underlie the familial renal cancer syndrome VHL disease [3]. In addition to CCRCCs, patients with VHL disease are also predisposed to phaeochromocytomas, haemangioblastomas of the central nervous system and retina and cysts affecting a variety of organs including the kidney and pancreas [2]. Although much has been learnt about *VHL *in recent years, its tumour suppressor function is still not fully understood.

**Figure 1:**
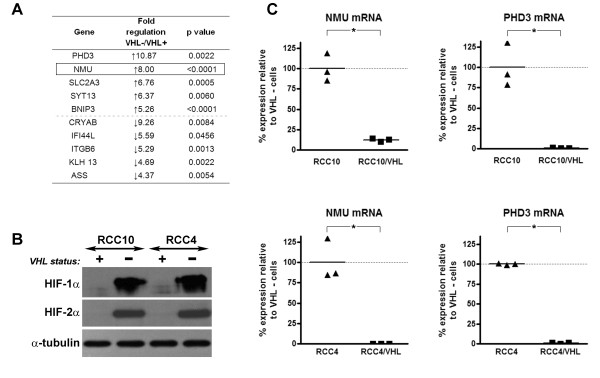
***VHL *regulates expression of Neuromedin U**. **A) **RCC10 retroviral cell pools infected with empty vector or expressing wild-type *VHL *were prepared as described previously [7]. Cells were cultured for 48 hours and RNA expression analysed using an Affymetrix U133 Plus 2.0 human gene expression array. Microarray analysis identified differentially expressed genes by comparing expression levels in *VHL *defective cells to expression in *VHL *expressing cells. The five most highly upregulated and down-regulated genes, including NMU are listed in 1a. **B) **Protein lysates were prepared from *VHL *defective RCC10 and RCC4 cell lines and sub-lines stably expressing *VHL *and samples run on a SDS Page gel. Membranes were probed with HIF-1α (BD, San Diego, CA), HIF-2α (Novus Biologicals, Littleton, CO) and α-tubulin (Sigma, St. Louis, MO). In the absence of VHL both HIF-1α and HIF-2α are clearly detected, however in the presence of VHL levels of both HIF-1α and HIF-2α are suppressed. Equal levels of α-tubulin confirm equivalent loading. **C) **Real-time RT PCR analysis for NMU and the HIF target gene PHD3 was performed using SYBR-Green PCR Master Mix (Abgene, Epsom, United Kingdom). Data presented here has been normalised to expression of β-actin. Cell lines expressing *VHL *show marked suppression of NMU and PHD3 compared to *VHL *defective cell lines (*p < 0.01, using student's T test). (Primers: NMU For: 5'-CCTCAAGGATTACAGCCTG-3', NMU Rev: 5'-GTTCCTGAGGCTTTGGTAG-3; PHD3 For F 5'-GATGCTGAAGAAAGGGC-3', PHD3 Rev R 5'- CTGGCAAAGAGAGTATCTG-3'; β-actin For 5'-CCCAGAGCAAGAGAGAGG-3', β-actin Rev 5'-GTCCAGACGCAGGATG-3').

There has been considerable success in developing new treatments for CCRCC that target aspects of the pathways related to loss of *VHL *function [1]. As a strategy to identify further potential targets, we examined the effect of re-expressing *VHL *in RCC10 renal cancer cells [4]. This cell background is attractive because re-expression of *VHL *alone restores many aspects of normal epithelial cell behaviour, including formation of tight junctions [5,6], adherens junctions [7,8] and a primary cilium [9-11]. Three separate pools of RCC10 *VHL *defective CCRCC cells were transduced with retroviruses expressing wild-type *VHL*; in parallel three pools were transduced with an empty vector. A substantial number of genes showed highly significant differences in expression, including many known to be modulated by *VHL *status (Figure 1A).

The neuropeptide Neuromedin U (NMU) was selected as being of particular interest for the following reasons. First, it was amongst the most highly regulated genes and has not previously been identified as modulated by *VHL*. Second, it acts on two identified G-protein coupled receptors making it potentially pharmacologically tractable [12]. Third, it has been implicated in autocrine growth and epithelial to mesenchymal transition in cancer [13]. Fourth, as a secreted peptide NMU may offer potential as a circulating or urinary biomarker in CCRCC. Recently Ketterer *et al*. showed that serum levels of NMU decrease following pancreas resection of pancreatic cancer patients [14].

NMU is a potent neuropeptide which was originally discovered in the 1980's. In humans *NMU *gives rise to a biologically active icosapentapeptide (NMU-25); function is dependent on a highly conserved C terminal sequence which is subject to enzymatic amidation. Several biological functions have been ascribed to NMU including regulation of smooth muscle contraction, blood pressure and local blood flow, ion transport in the gut, stress responses, gastric acid secretion, nociception and feeding behaviour [12,15,16].

To confirm the effect of VHL status on NMU expression that we observed on microarray analysis of retrovirally transduced pools of RCC10 cells, we next examined NMU expression in RCC10 cells and a subline stably expressing wild-type *VHL *(RCC10/*VHL*). We also examined a second *VHL *defective renal cancer cell line and subline expressing VHL derived from a different patient (RCC4 and RCC4/*VHL*). As expected, VHL suppressed HIF-α protein levels (Figure 1B) and mRNA expression of the HIF target gene *PHD3 *(Figure 1C) in both cell backgrounds. Real time RT-PCR analysis showed NMU mRNA expression was markedly increased in the absence of functioning *VHL *in both RCC10 and RCC4 cells (Figure 1C).

Several different biochemical functions have been reported for VHL including regulation of microtubule stability, cell differentiation, cell motility, extracellular matrix assembly, JunB and atypical isoforms of protein kinase C [2]. However the most extensively studied function of VHL is regulation of HIF [17]. HIF is comprised of a constitutively active β subunit and an oxygen-regulated α subunit. In the presence of oxygen the α-subunit is hydroxylated by a group of prolyl hydroxylase domain (PHD) enzymes, leading to capture by VHL, ubiquitination and proteosomal destruction [17-19]. In hypoxia (or if *VHL *is inactivated) HIF α is stabilised. HIF is a master regulator of oxygen homeostasis and drives the transcriptional upregulation of >100 genes involved in processes such as glucose transport, glycolysis and angiogenesis, which collectively allow the adaption of the cells, tissues and organisms to low oxygenation [2,17,18].

**Figure 2:**
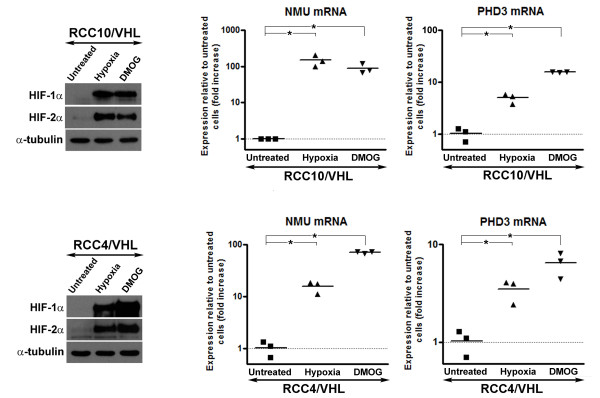
**Neuromedin U is upregulated by hypoxia and DMOG in *VHL *expressing CCRCC cell lines**. RCC10/*VHL *and RCC4/*VHL *cell were exposed to either 1% hypoxia in a hypoxic Galaxy R incubator (Biotech, Palo Alto, CA) or 1 mM dimethyl-oxalylglycine (DMOG; Frontier Bioscientific, Logan, UT) for 48 hours. Western analysis showed marked upregulation of both HIF-1α and HIF-2α in treated cell lines. Levels of NMU and the HIF target gene PHD3 were found to be significantly upregulated using real-time RT PCR analysis (*p < 0.01 using Student's t test).

To investigate whether activation of HIF mediates the high expression of NMU we exposed RCC10 cells stably expressing *VHL *to hypoxia as a means of activating HIF. Exposure of RCC10/*VHL *cells to hypoxia for 48 hrs resulted in a significant increase in mRNA levels of NMU compared to normoxic cultures (Figure 2). In a second approach, we activated HIF in the presence of *VHL*, using dimethyloxalylglycine (DMOG), a cell permeable 2-oxyglutarate-dependent dioxygenase inhibitor which inhibits the PHD enzymes. Treatment with DMOG resulted in a striking induction of both *NMU *mRNA compared to untreated cells (Figure 2). These results would be consistent with HIF activation mediating increased NMU expression in *VHL *defective CCRCC cells.

**Figure 3:**
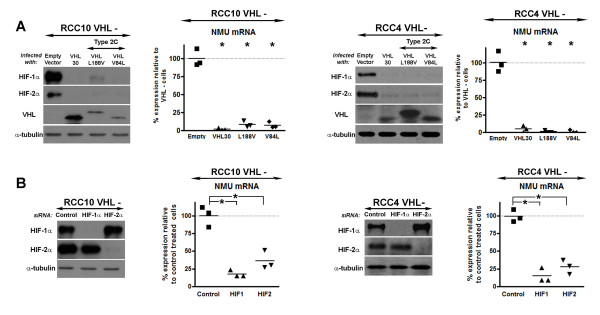
**Suppression of HIF-α subunits in RCC10 and RCC4 CCRCC cell lines decreases NMU expression**. **A) ***VHL Type 2C mutants suppress HIF and NMU*: Retroviral RCC10 and RCC4 cell pools expressing either wild-type *VHL *(*VHL*30), a type 2C mutant *VHL *(either *VHL *L188V or *VHL *V84L) or an empty vector were prepared as described previously [7]. Expression of VHL was confirmed by Western blotting. Analysis of HIF-α subunit expression showed that levels of both HIF-1α and HIF-2α were suppressed in cell pools expressing either wild-type VHL or type 2C mutant VHL. NMU expression was significantly decreased in RCC10 and RCC4 cell lines expressing either wild-type or type 2C mutant VHLs, but not an empty vector control. **B) ***Suppression of HIF via siRNA of HIF-α subunits results in down-regulation of NMU: *RCC10 and RCC4 cells were transfected with a control siRNA (firefly luciferase) or siRNA oligos targeting HIF-1α or HIF-2α using LipofectAMINE 2000 (Invitrogen, Carlsbad, CA). (Oligos: Firelfly Luciferase 5'-CGUACGCGGAAUACUUCGAdTdT-3' (sense), 5'-AAGCUAAAGGUACACAAUUdTdT-3' (antisense); HIF-1α 5'-CUGAUGACCAGCAACUUGAdTdT-3' (sense), 5'-UCAAGUUGCUGGUCAUCAGdTdT-3' (antisense); HIF-2α 5'-CAGCAUCUUUGAUAGCAGUdTdT-3' (sense), 5'-ACUGCUAUCAAAGAUGCUGdTdT-3' (antisense). Cells were plated in duplicate and harvested 48 hours post transfection for either RNA or protein. Western blotting for HIF-1α and HIF-2α confirmed selective and efficient knock-down of the targeted HIF-α subunit. Real-time RT PCR showed that HIF-α knock-down significantly suppressed NMU expression in both RCC4 and RCC10 cells. *p < 0.01, using Student's t test.

To further investigate the ability of HIF to mediate NMU expression we studied the effect of *VHL *missense mutants, L188V and V84L. These single amino acid substitutions are associated clinically with phaeochromocytoma, but not with other tumours (type 2C *VHL *disease) and retain the ability to regulate HIF [20]. Analysis by real time RT-PCR showed a marked mRNA suppression of NMU in cell pools expressing either wild type *VHL *or type 2C mutant *VHL*, in both RCC10 and RCC4 cells (Figure 3A). The concordance between the ability of VHL mutants to suppress both HIF and NMU levels supports the notion that HIF activation underlies the *VHL *defective phenotype. Next we used a direct genetic approach to determine the role of HIF. RCC10 and RCC4 *VHL *defective cells were transfected with previously validated siRNAs to selectively suppress either HIF-1α or HIF-2α, or with a control siRNA. Knock-down of either HIF-α isoform resulted in decreased expression of NMU in both cell backgrounds (Figure 3B). Taken together these findings provide strong evidence that *VHL *regulates NMU expression in renal cancer cells via HIF.

Next we asked whether NMU contains a HIF binding site, known as a hypoxia response element (HRE). A 12 kb segment upstream of the transcriptional start site and the first intron (3 kb) were analysed. Although this region contains several candidate HREs, none were found to be responsive to HIF activation as assessed by increased luciferase reporter activity. There are three possible explanations for this. First, the region may contain a functional HRE but the transfection assays did not provide requisite conditions to detect this. This possibility is lessened because we used different approaches to activate HIF (DMOG or co-transfection with a plasmid expressing constitutively active HIF), different cell lines, and all experiments were accompanied by controls using an HRE which confirmed a high amplitude HIF response. Second, there may be an HRE which lies outside this region; a precedent for this is the prototypical HRE which actually lies 3' to the *EPO *gene [21]. Third, the effect of HIF may be indirect.

**Figure 4:**
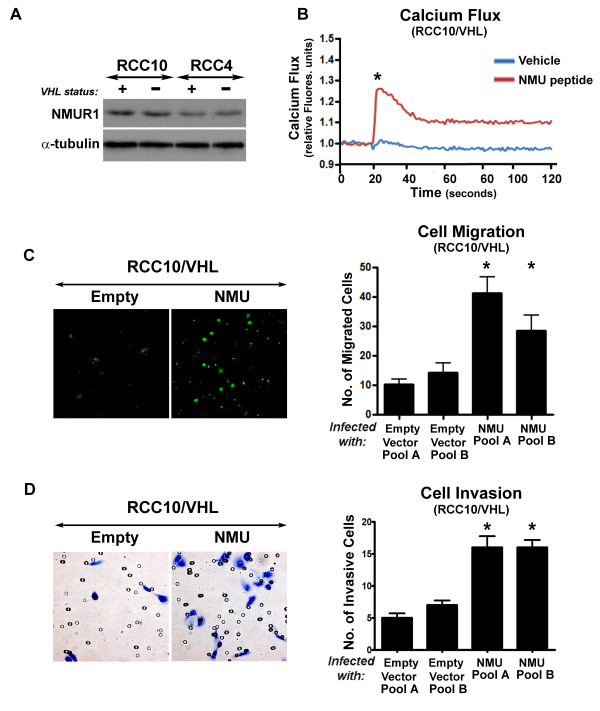
**The NMU pathway is functionally active in renal cancer cells and contributes to a migratory and invasive phenotype**. **A) **Western blotting showed that NMUR1 is expressed in CCRCC cells. **B) ***NMU peptide induces a calcium flux in renal cancer cells: *RCC cell cultures were loaded with a calcium sensitive dye (fluo-4 AM; Invitrogen, Carlsbad, CA) and washed with HBSS. A fluorometric imaging plate reader (FLIPR; Molecular Devices, Sunnyvale, CA) recorded fluorescence (λEX = 488 nm, λEM = 540 nm) before and after robotic addition of 60 μL of NMU peptide (final concentration = 300 μM) or a vehicle control. A representative trace from RCC10/*VHL *cells is shown. RCC10, RCC4 and RCC4/*VHL *cells all showed similar results. Significance was determined by analysing peak fluorescence induction in NMU treated and untreated cells from five independent experiments. **C & D) ***Constitutive expression of NMU promotes cell migration and invasion: *Retroviral cell pools expressing either NMU or an empty vector were prepared in RCC10/*VHL *cells. Cells were serum-starved, fluorescently labelled (migration assay only) and plated onto the upper compartment of FluoroBlok transwell migration chambers (**C**) or matrigel BioCoat transwell invasion chambers (**D**). Media with 5% FCS was added to the lower compartment. After 9 hours (migration assays) or 24 hours (invasion assays) cells were fixed. Invasion membranes were stained with Giemsa. Cells were counted in five fields. RCC10/*VHL *cells expressing NMU showed significantly more migration and invasion than empty vector control cells (*p < 0.05, Student's t test, three independent experiments).

NMU is believed to act mainly in a paracrine fashion, and autocrine signalling has been suggested in several cancer settings [13]. To determine whether renal cancer cells might respond to NMU we first examined expression of the receptors NMUR1 and NMUR2. Western analysis showed that NMUR1 was expressed in both the RCC4 and RCC10 cell backgrounds and that there was no substantial effect of *VHL *status on expression levels (Figure 4A). Expression of NMUR2 was not detected (data not shown). Previous studies have reported that NMU receptor binding triggers the mobilisation of intracellular calcium [13]. Renal cancer cells and sublines expressing *VHL *(RCC10, RCC4, RCC10/*VHL*, RCC4/*VHL*) were loaded with a calcium sensitive fluorescent dye prior to treatment with exogenous NMU peptide. Addition of NMU caused a sharp calcium influx in all cell lines, regardless of *VHL *status, whereas addition of vehicle alone caused no significant change. A representative FLIPR trace is shown in Figure 4B. Given that these experiments show that an NMU response pathway is present in renal cancer cells, we next investigated the potential functional role of NMU in these cells.

Proliferation and tissue invasion and metastasis are two hallmarks of cancer that have been associated with NMU expression in other cell types [14,22,23]. First we tested the effect of NMU on proliferation. Exogenous NMU peptide was added to RCC10/*VHL *cells; no effect on cell growth was observed (data not shown). Next we tested the ability of NMU to promote migration and invasion of renal cancer cells. RCC10/*VHL *retroviral cell pools expressing either an empty vector or NMU were prepared and NMU expression was verified by real-time RT-PCR. To assess migration, cells were serum starved for 24 hours, fluorescently labelled and then plated onto the upper section of a transwell chamber in serum free media. The number of cells that migrated through the membrane was recorded. Invasion assays were performed by plating serum-starved cells onto matrigel coated transwell chambers in serum free media. After 24 hours, cells were removed from the top layer and invasive cells on the bottom layer were fixed, stained with Giemsa and counted. Data from three independent experiments and representative photomicrographs are shown in Figure 4C and 4D. Thus expression of NMU was found to significantly enhance both cell migration and invasion of RCC10/*VHL *cells.

Next, we searched the Gene Expression Omnibus database (GEO, http://www.ncbi.nlm.nih.gov/geo/) for publicly available microarray datasets comparing gene expression in normal human kidney tissues to CCRCC tissues. Three relevant datasets were identified. Analysis of data from Gumz *et al*. showed a > 2 fold upregulation of NMU in 5/10 pairs of matched normal kidney and tumour tissue from patients with sporadic CCRCCs (Additional File 1a) [24]. Analysis of two larger, independent datasets comparing unmatched normal kidney and CCRCC tissues, GSE15641 and GSE14994, also showed > 2 fold upregulation of NMU in 46.9% and 11.9% of CCRCCs, respectively, compared to the mean expression in normal kidney (Additional File 1b and 1c) [25,26]. These results are consistent with NMU expression being substantially increased in a subset of human CCRCCs.

Loss of functional *VHL *has previously been shown to enhance migration and invasion [27]. Here we demonstrate an autocrine NMU pathway in renal cancer cells that likely contributes to promoting migration of *VHL *expressing cells. This adds to evidence for a role of NMU in diverse cancers including bladder carcinoma [22], ovarian carcinoma [28], lung cancer [23] and acute myeloid leukaemia [13]. It also raises the possibility that HIF activation, for example at altitude, could increase physiological NMU signalling in the brain causing loss of appetite.

## List of abbreviations

CCRCC: clear cell renal cell carcinoma; FLIPR: fluorometric imaging plate reader; GEO: Gene Expression Omnibus; HIF: hypoxia-inducible factor; HRE: hypoxia response element; DMOG: dimethyl-oxalylglycine; NMU: Neuromedin U; PHD: prolyl hydroxylase domain; VHL: von Hippel-Lindau.

## Competing interests

SKH, MA, DS and MAE declare that they have no competing interests. PHM is a Director and shareholder in ReOx Ltd.

## Authors' contributions

SKH prepared the retroviral expression plasmid encoding NMU and retroviral cell pools used throughout the study, produced most of the experimental data and drafted the initial manuscript. MAE contributed to the study design and prepared retroviral vectors encoding *VHL *type 2C mutants. DS assisted with addressing reviewer comments. MA provided input on experimental strategy and study design. PHM co-ordinated the study and wrote the final manuscript. All authors contributed to study design, analysis and interpretation of data and read and approved the final manuscript.

## Supplementary Material

Additional File 1 **External data sets show a subset of clear cell renal carcinomas with upregulation of NMU compared to normal kidney**. Publicly available microarray datasets comparing expression in normal human kidney tissue to CCRCC tissues were interrogated for NMU expression. **A) **A scatter plot showing NMU expression in matched normal and tumour tissue samples from patients with sporadic CCRCCs (GDS2880; [24]). 5/10 pairs show > 2 fold upregulation of NMU (data points shown in red). **B), C) **Scatterplots show fold upregulation of NMU in normal kidney and CCRCC tissue samples compared to the mean expression level detected in normal kidneys in two external datasets, GSE15641 [26] and GSE14994 [25]. Data points in red show > 2 fold upregulation (GSE15641, 15/32 (46.9%) CCRCC samples show >2 fold upregulation; GSE14994, 7/59 (11.9%) of CCRCC samples show >2 fold upregulation). Normalised data available from GEO was used for all analyses. Click here for file
