# Supplementary figures and images for: Inactivation of the von Hippel-Lindau tumour suppressor gene induces Neuromedin U expression in renal cancer cells
Source: Mol Cancer. 2011 Jul 26;10:89. doi: 10.1186/1476-4598-10-89 (PMC3155908; doi:10.1186/1476-4598-10-89)

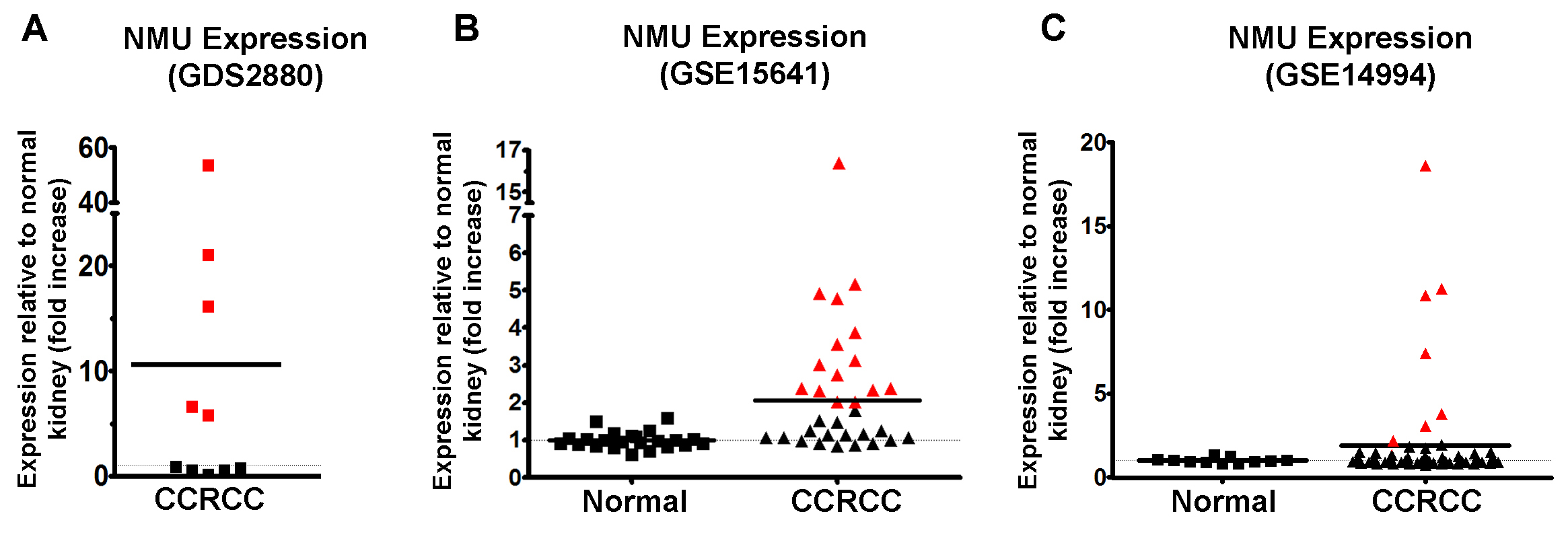

Supplement: Additional File 1 — External data sets show a subset of clear cell renal carcinomas with upregulation of NMU compared to normal kidney. Publicly available microarray datasets comparing expression in normal human kidney tissue to CCRCC tissues were interrogated for NMU expression. A) A scatter plot showing NMU expression in matched normal and tumour tissue samples from patients with sporadic CCRCCs (GDS2880; [24]). 5/10 pairs show > 2 fold upregulation of NMU (data points shown in red). B), C) Scatterplots show fold upregulation of NMU in normal kidney and CCRCC tissue samples compared to the mean expression level detected in normal kidneys in two external datasets, GSE15641 [26] and GSE14994 [25]. Data points in red show > 2 fold upregulation (GSE15641, 15/32 (46.9%) CCRCC samples show >2 fold upregulation; GSE14994, 7/59 (11.9%) of CCRCC samples show >2 fold upregulation). Normalised data available from GEO was used for all analyses. [file 1476-4598-10-89-S1.JPEG]
